# Chitosan mitigates water stress in cowpea plants through modulation of growth, homeostasis, and antioxidant activities

**DOI:** 10.3389/fpls.2025.1591920

**Published:** 2025-06-06

**Authors:** Rayanne Silva de Alencar, Priscylla Marques de Oliveira Viana, Guilherme Felix Dias, Semako Ibrahim Bonou, Leticia Diniz Ribeiro, Yngrid Mikhaelly Lourenço de Araujo, Igor Eneas Cavalcante, Hermes Alves de Almeida, Pedro Roberto Almeida Viégas, Alberto Soares de Melo

**Affiliations:** ^1^ Posgraduate Program in Agricultural Sciences, State University of Paraiba, Campina Grande, Paraíba, Brazil; ^2^ Academic Unit of Agronomic Engineering, Federal University of Campina Grande, Campina Grande, Paraíba, Brazil; ^3^ Department of Biology, Paraíba State University, Campina Grande, Paraíba, Brazil; ^4^ Departament of Agronomy, Federal University of Sergipe, São Cristovão, Sergipe, Brazil

**Keywords:** chitosan, *Vigna unguiculata* (L.) Walp., biopolymer, water restriction, antioxidant mechanism, osmoprotection

## Abstract

**Introduction:**

Climate change and population growth increase food demand, especially in semi-arid regions. Water deficit affects cowpea productivity, but foliar application of chitosan can improve its tolerance, stimulating antioxidant activity and growth. This study analyzed chitosan application in cowpea (cv. BRS Olhonegro) under different irrigation levels, seeking alternatives to enhance productivity.

**Methods:**

The experiment was conducted in a growth chamber using a completely randomized design. Three concentrations of chitosan (0, 50, and 75 mg L^-1^) were tested at varying irrigation depths (W100: 100% and W50: 50% replacement of plants evapotranspiration). At phenological stages V5 and V7, several assessments were carried out, including water status and membrane damage evaluation, leaf pigment analysis, enzymatic and non-enzymatic antioxidant activity measurement, growth evaluation, and water use efficiency determination.

**Results:**

At the V5 development stage, the 50 mg L^-1^ concentration positively influenced the relative water content, superoxide dismutase enzyme activity, proline content and total shoot dry mass. In addition, it reduced intracellular electrolyte leakage. At the V7 stage, a 75 mg L^-1^ concentration was particularly effective in reducing the impact of water restriction, mainly by increasing the activity of ascorbate peroxidase, proline and chlorophyll in the BRS Olhonegro cultivar.

**Discussion:**

In summary, chitosan application mitigated the adverse effects of water stress in cowpea by maintaining water balance, preserving photosynthetic pigments, enhancing antioxidant mechanisms, and providing osmoprotection to the crop. This study highlights chitosan's potential as a cost-effective and environmentally friendly strategy to increase cowpea resilience to drought, an essential crop for food security in semi-arid regions.

## 1 Introduction

Global climate change has led to changes in the spatial and temporal distribution of rainfall, resulting in water deficits in the soils of agroecosystems, mainly impacting tropical and subtropical regions, where rainfall volumes were no longer sufficient to meet crop water demands. These unfavorable conditions reduce plant growth and agricultural production, putting the food and nutritional security of populations at risk (Zhang et al., 2021; Mekonnen et al., 2022). A comprehensive understanding of plant plasticity is crucial for developing genetically improved genotypes that thrive in changing environmental conditions. In this context, cowpea (*Vigna unguiculata* (L.) Walp.) is a Fabaceae that stands out as a promising option due to its socioeconomic importance as a subsistence crop and its qualities developed for farmers, mainly in the North and Northeast regions of Brazil (Oliveira et al., 2023; Alencar et al., 2024).

Cowpea is extensively grown in sustainable agriculture due to its moderate tolerance to water stress, high resistance to salinity, and adaptability to a wide temperature range (18 to 34°C). Its seeds are also highly nutritional (Melo et al., 2022; Jayawardhane et al., 2022). In semiarid regions like the Brazilian Northeast, where water stress is prevalent cowpea plant cultivation encounters additional challenges.

Recent studies show that this type of stress can reduce the leaf area and biomass of cowpea, as well as decrease stomatal conductance and transpiration, which results in decreased photosynthetic rates and increased levels of reactive oxygen species (ROS), which affect the integrity of cell membranes by increasing lipid peroxidation (Hidangmayum and Dwivedi, 2023; Cavalcante et al., 2024). However, it is noteworthy that cowpea can circumvent these deleterious effects by activating defense mechanisms, such as: osmotic adjustment through the accumulation of compatible solutions, which helps maintain water absorption under drought conditions; as well as enzymatic and non-enzymatic antioxidant systems, responsible for eliminating ROS, keeping them at levels compatible with the proper functioning of cellular metabolism (Alencar et al., 2024).

It is essential to explore technologies, such as elicitor substances, that improve water use efficiency and optimize metabolic pathways to promote water stress tolerance in cowpea plants. Chitosan, a natural biopolymer derived from chitin, is a promising option that acts as a biostimulant and elicitor in sustainable agriculture, mainly because it is non-toxic, diodegradable and biocompatible (Alenazi et al., 2024).

Chitosan is known to help plants resist water deficit and adapt to adverse conditions, making it a valuable tool to increase plant resilience (Alenazi et al., 2024). This compound is known to increase water use efficiency, trigger defense mechanisms against oxidative stress, and facilitate osmotic adjustments in plants (Almeida et al., 2020; Makhlouf et al., 2022; Hidangmayum and Dwivedi, 2023). However, there is limited research on the foliar application of chitosan in cowpea under water restriction conditions, highlighting the need for further studies. To this end, this study evaluated the efficacy of foliar application of chitosan as a mitigator of water deficit stress in cowpea cv. BRS Olhonegro under two irrigation levels.

## 2 Materials and methods

The experiment was conducted in a Fitotron growth chamber at the Botanical Garden of the State University of Paraíba (UEPB). Biochemical analyses were performed at the Ecophysiology of Cultivated Plants Laboratory (ECOLAB) at UEPB, situated at Três Marias Integrated Research Complex (Campus I) in Campina Grande, PB. The location coordinates are 07°13’50’’ latitude, 35°52’52’’ longitude, and 551 m altitude.

### 2.1 Plant material

**Figure 1:**
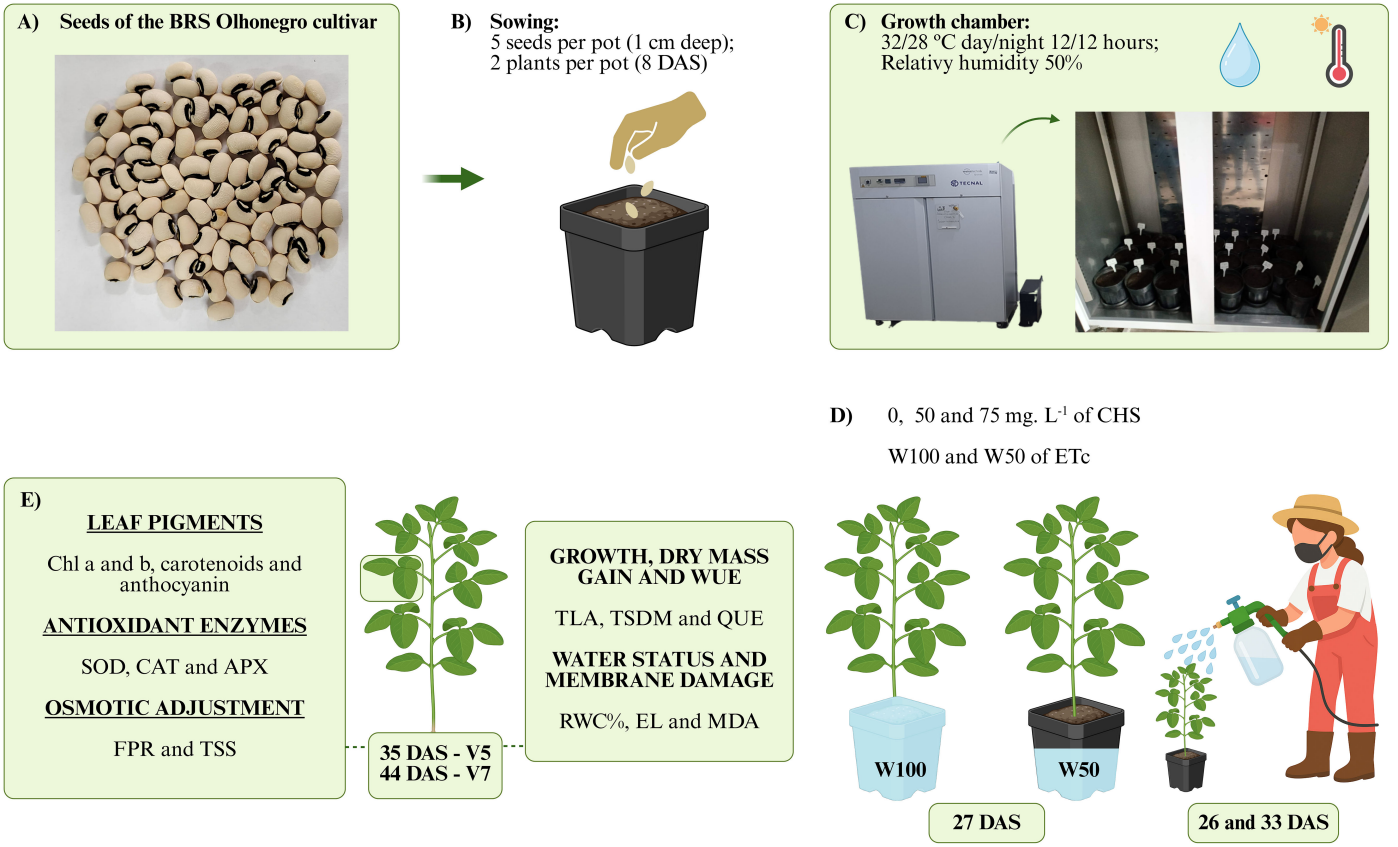
Diagram of the experiment and treatment application. **(A)** seeds of the BRS Olhonegro cultivar, **(B)** sowing, **(C)** arrangement of pots in a Fitotron growth chamber, **(D)** application of QS concentrations at 26 and 33 days after sowing (DAS) and differentiation of irrigation depths (27 DAS), **(E)** variables analyzed at phenological stages V5 and V7. This figure was created using PowerPoint.

The experiment used the cowpea variety BRS Olhonegro, considered early, with maturity occurring 61 to 70 days after sowing. This characteristic is ideal for semiarid regions, due to short rainy periods. For this purpose, seeds of this variety were obtained from the germplasm bank of the Brazilian Agricultural Research Corporation (Embrapa Meio-Norte) (**Figure 1**).

### 2.2 Treatments applications and experimental design

The experimental design was completely randomized (CRD) in a 3 x 2 factorial scheme, with three concentrations of chitosan (CHS) and two irrigation levels. Chitosan concentrations of 0, 50, and 75 mg L^−^¹, adapted from Jiao et al. (2024), were applied to the plants at 26 and 33 days after sowing (DAS). At 27 DAS, one day after the first chitosan application, the plants were subjected to irrigation levels of 50% (W50), corresponding to 50% of the crop evapotranspiration water replacement (ETc), and 100% of ETc (W100), to evaluate the effects of chitosan under both optimal and stress conditions. The combination of the two factors resulted in six treatments, with four replicates per treatment, totaling 24 experimental units. Chitosan (C_4_H_11_O_4_N) was purchased from Grupo Patense. An 8% solution was prepared by dissolving 80 g of chitosan in 80 g of acetic acid, diluted in filtered, chlorinated, potable water to a final volume of 1 L. This solution was then diluted to concentrations of 50 and 75 mg L^−^¹ of chitosan. The seeds were carefully sieved to remove any damaged or malformed seeds. After sieving, the seeds were disinfected with 1% sodium hypochlorite for 3 minutes, then washed and dried (Carvalho and Carvalho, 2009). Five seeds were sown at a depth of 1.0 cm in 3.6 L polyethylene pots filled with 3.5 kg of soil (**Figure 1**).

The soil used in the experiment had the following characteristics: sand (83.62%), silt (6.02%), clay (10.26%), soil density (1.47 g cm^-3^), particle density (2.60 g cm^-3^), porosity (43.46%), calcium (2.36 cmol_c_ kg^-1^), magnesium (2.71 cmol_c_ kg^-1^), sodium (0.13 cmol_c_ kg^-1^), potassium (0.46 cmol_c_ kg^-1^), súlfur (5.66 cmol_c_ kg^-1^), hidrogen (cmolc kg^-1^), aluminium (0.20 cmol_c_ kg^-1^), organic matter (24.74 g kg^-1^), and pH (5.55).

The pots were irrigated to reach the pot water capacity and then transferred to the Fitotron growth chamber model SGC120 from Weiss Technik. Set the temperature to fluctuate between 32°C during the day and 28°C at night for 12 hours each. The humidity was maintained at 50%. The daily routine was performed using the pot weighing method, according to Silva et al. (2020). At 8 DAS the seedlings were thinned, leaving only two per pot (**Figure 1**).

On the 26th DAS, topdressing fertilization was applied using DripSol MAP (Monoammonium Phosphate: N-2%, P_2_O_5_-65%, and K_2_O-0%) and KCl (60%) via fertigation with 1.33 g and 1.1 g, respectively, diluted in 13 L of water. On the 27th DAS, the fungicide Amistar^®^ was applied (10 mg), diluted in 1,0 L of water, and 1 mL of adhesive spreader according to the manufacturer’s recommendations.

### 2.3 Variables analyzed

The indicators of water status and membrane damage, chloroplast pigments, antioxidant mechanism activity, indicators of osmotic adjustment, growth, dry mass gain, and water use efficiency (WUE) were evaluated at 33 and 44 DAS (phenological stages V5 and V7), in order to evaluate the effect of treatments throughout crop development, as well as after each chitosan application.

#### 2.3.1 Indicators of water status and membrane damage

The methodology outlined by Brito et al. (2011) and Alencar et al. (2024) was employed to analyze the relative water content (RWC%) and intracellular electrolyte leakage (EL%). Ten leaf discs measuring 5 mm in diameter for RWC and EL, respectively, were obtained using a copper perforator.

##### 2.3.1.1 Relative water contente

The relative water content (RWC%) was determined by weighing leaf discs to obtain the fresh weight (DFM). The discs were placed in aluminum capsules with 10 mL of distilled water, sealed with plastic wrap, and left at room temperature. After 24 hours, excess water was removed with paper, and the discs were weighed to obtain the turgid weight (DTM). Afterward, the leaf discs were placed in paper bags, identified, and dried in an oven with forced air circulation at 60°C for over 48 hours to obtain the dry mass of the discs (DDM). RWC% was calculated using the following Equation 1.


(1) $$\text{RWC}\;\left(\%\right)=\;\left[\left(\frac{DFM-DDM}{DTM-DDM}\right)\right]*100$$

##### 2.3.1.2 Electrolyte leakage

Electrolyte leakage (EL%), a measure of intracellular membrane damage, was assessed by placing the disks in test tubes with 10 mL of distilled water. The tubes were sealed and rested for 24 hours. After this period, the electrical conductivity of the solution was measured in the test tubes (initial conductivity, Xi) using a portable conductivity meter (WATERPROOF). The tubes were sealed and exposed to 100°C for 60 minutes in a water bath. After cooling to room temperature, the final electrical conductivity (Xf) of the solution was measured. The intracellular electrolyte leakage percentage was calculated using the following Equation 2.


(2) $$EL\;\left(\%\right)=\;\left(\frac{Xi}{Xf}\right)*100$$

##### 2.3.1.3 Water use efficiency

The water use efficiency (WUE) was calculated from the proportion between the weight of total dry matter of the plant shoot (TDMS) and plant water consumption (ETc) where: WUE: water use efficiency, kg ha^-1^ mm^-1^, TDMS: total dry matter of shoots, kg ha^-1^. To obtain the value of dry mass per hectare, according to Silva (2023), an area of 0.5 m2 with 15 plants was measured obtaining a total value of 300,000 plants ha^-1^, ETc: total water consumption, mm Silva et al. (2013), according to the Equation 3.


(3) $$WUE=\frac{TDMS}{ETc}$$

##### 2.3.1.4 Lipid peroxidation

Lipid peroxidation was determined from the amount of malonaldehyde (MDA) produced by thiobarbituric acid (TBARS) assay, following a modified protocol based on Cakmak and Horst (1991). Approximately 0.2 g of fresh tissue was macerated with 2 mL of 0.1% trichloroacetic acid (TCA). Aliquots containing 0.5% thiobarbituric acid (TBA, w/v) and 10% TCA were then incubated at 90°C for 20 minutes, followed by rapid cooling in an ice bath to stop the reaction. After centrifugation to clarify the sample and remove any interfering substances, the net absorbance values were determined by subtracting the nonspecific absorbance at 600 nm. Lipid peroxidation, quantified as malondialdehyde content, was expressed in nmol g FM^-1^.

#### 2.3.2 Leaf pigments

Chlorophyll a and b, carotenoids, and anthocyanins content were quantified following the method proposed by Sims and Gamon (2002). Fresh material (0.2 g) was weighed and ground with 3 mL of ice-cold 80% acetone/Tris-HCl buffer solution (80:20 v:v, pH 7.8) in a light-protected environment. The absorbance of the solution was measured using an AKSO model UV1720 spectrophotometer at 663, 646, 573, and 470 nm. The absorbance values were then converted to mg/100 g FM using specific Equations 4, 5, 6 and 7.


(4) $$\text{Chlorophyl a}=0.01373A_{663}-0.000897A_{573}-0.003046A_{646}$$

(5) $$\text{Chlorophyll b}=0.02405A_{646}-0.004305A_{573}-0.005507A_{663}$$

(6) $$\text{Anthocyanin}=\;0.08173A_{573}-0.00697A_{646}-0.002228A_{663}$$

(7) $$\text{Carotenoids}:\;\frac{(\left(A_{470}-\left(17.1\;\times \;\left(\text{chlorophyll a}+\text{chlorophyll b}\right)-9.479\;\times \text{ Anthocyanin}\right)\right)}{119.26}$$

#### 2.3.3 Antioxidant mechanism activity

The antioxidant mechanism was evaluated by measuring the enzymatic activities of superoxide dismutase (SOD, UA gFM^-1^), catalase (CAT, μmol of H2O2 min^-1^ gFM^-1^) and ascorbate peroxidase (APX, μmol of asc min^-1^ gFM^-1^) in a spectrophotometer (model Nova 2000 UV).

Initially, 200 mg of fresh material was ground with 2 mL of 50 mM phosphate buffer (pH 7.0) along with ascorbic acid (0.1 mM), EDTA (0.1 mM), and polyvinylpyrrolidone (5%). The extracts were then centrifuged at 20,000 G for 20 minutes at 4°C, and the supernatant was transferred to 2.5 mL plastic tubes (Eppendorf).

##### 2.3.3.2 Superoxide dismutase activity

To determine SOD activity, a mixture composed of 0.3 mL of 130 µM methionine, 0.1 mL of 2250 µM p-nitro blue tetrazolium (NBT), 0.1 mL of 3 µM EDTA, 0.2 mL of riboflavin, 0.75 mL of deionized water, and 1.5 mL of 50 mM sodium phosphate buffer at pH 7.8 plus 100 µL of the enzyme extract. The absorbance of the reaction without the enzyme extract was measured. SOD activity was determined based on the photoreduction inhibition capacity of NBT Beauchamp and Fridovich (1971).

##### 2.3.3.3 Catalase activity

Catalase activity was measured by adding 150 µL of the enzyme extract to 1950 µL of potassium phosphate buffer (100 mM, pH 7.5) of the solution with deionized water (150 µL of the extraction buffer and 750 µL of hydrogen peroxide) (Kar and Mishra, 1976). The enzymatic activity was observed by monitoring the decrease in absorbance at 240 nm.

##### 2.3.3.4 Ascorbate peroxidase activity

The APX activity was determined by adding 100 µL of the extract into 2.7 mL of the reaction medium containing potassium phosphate buffer (50 mM,pH 6) and ascorbic acid (0.8 mM).The reaction started by adding 200 µL of hydrogen peroxide H_2_O_2_ (2 mM). Enzyme activity was assessed by monitoring the decrease in absorbance at 290 nm (Nakano and Asada, 1981).

#### 2.3.4 Osmotic adjustment indicators

The free proline content (FPR μmol g^-1^ of fresh matter) was determined using a colorimetric method based on the procedure outlined by Bates et al. (1973) with modifications described by Bezerra Neto and Barreto (2011). Initially, 250 mg of fresh matter sample was macerated in 5 mL of 3% sulfosalicylic acid and then centrifuged at 2,000 rpm for 10 minutes. The resulting supernatant was collected in 2.5 mL tubes for subsequent measurement of proline concentration.

Total soluble sugars (TSS) were quantified using a quote from 200 mg of fresh leaflet mass and 100 mg of fresh root mass. Initially, these samples were macerated in 2 mL of 80% (v/v) ethanol. This extract was added to Eppendorf tubes (2 mL capacity) and placed in a water bath (60°C) for 30 minutes, then transferred to a centrifuge (2000×g) to obtain and collect the supernatant. After collecting the supernatant, an additional 2 mL of ethanol (80%) was added to the same tubes for a second extraction. The tubes were then heated in a water bath and transferred to a centrifuge. The supernatants from both extractions were combined in Falcon tubes and stored in Eppendorf tubes, resulting in a total of 4 mL of extract per sample. The concentrations were determined using the phenol-sulfuric method according to Dubois et al. (1956).

#### 2.3.5 Growth and dry weight gain measurements

The total leaf area (TLA) and total shoot dry mass (TSDM) were evaluated following the method according to Cavalcante et al. (2024). Total leaf area was determined by scanning the leaflets using a Motorola One cell phone with a 13 MP + 2 MP + 2 MP macro rear camera and laser focus on a two-centimeter scale. The images were labeled accordingly, and the leaf area was measured in cm^2^ using ImageJ software (Version 1.54h).

Plants were placed separately in identified paper bags to dried in an oven with forced air circulation at 70°C for 72 hours to obtain total shoot dry mass. After this period, plant materials were weighed on an analytical balance (e = 0.0001 g).

### 2.4 Statistical analysis

The data were analyzed employing the Shapiro-Wilk normality test Shapiro and Wilk (1965). Once normality assumptions were met, mean comparison tests (Tukey, *P* ≤ 0.05) were conducted for chitosan concentrations, and independent t-tests (*P* ≤ 0.05) were performed for water replacement levels (50% and 100%) using Sisvar 5.6 (Ferreira, 2019). The Pearson correlation matrix was generated using R Studio’s ggcorrplot package version 4.2.3.

## 3 Results

### 3.1 Indicators of water status and membrane damage

**Figure 2:**
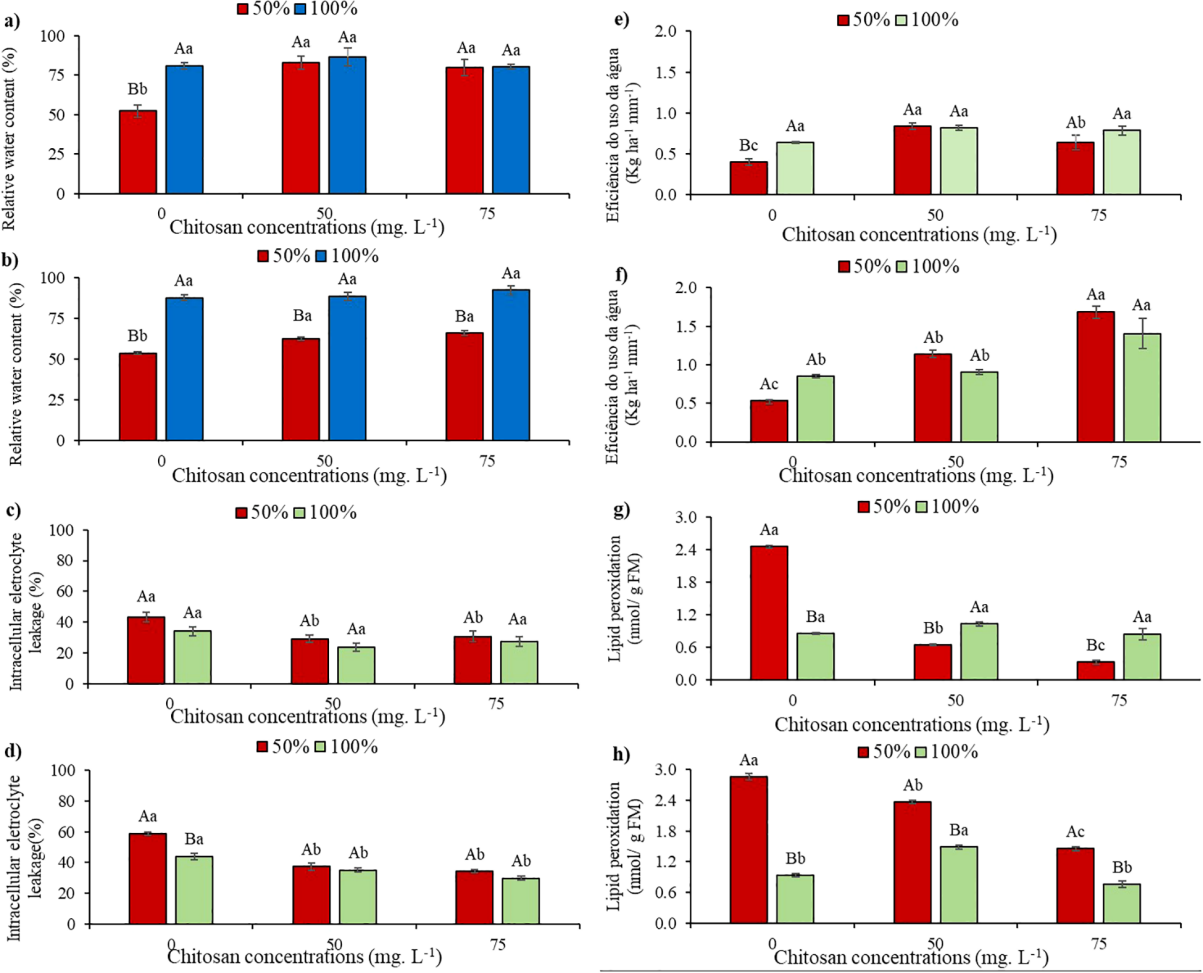
Relative water content at phenological stages V5 **(a)** and V7 **(b)**; intracellular electrolyte leakage at phenological stages V5 **(c)** and V7 **(d)**; water use efficiency at phenological stages V5 **(e)** and V7 **(f)** and lipid peroxidation at phenological stages V5 **(g)** and V7 **(h)** of cowpea cv. BRS Olhonegro, conditioned to two irrigation depths (50% and 100% water replacement from crop evapotranspiration) and three chitosan concentrations (0, 50, and 75 mg. L^-1^). Capital letters differentiate irrigation depths within each chitosan concentration (t-Student *P* ≤ 0.05) and lowercase letters differentiate chitosan concentrations within each irrigation depth (Tukey *P* ≤ 0.05).

Under water restriction, the relative water content (RWC,%) decreased by 35.3% and 38.7% at phenological stages V5 and V7, respectively, compared to the control water levels without chitosan (**Figures 2a, b**).

At stages V5 and V7, and under water level W50, there were increases of 58.5% and 16.4% (50 mg L^-1^) and 52.6% and 22.5% (75 mg L^-1^), respectively, compared to plants without chitosan application (**Figures 2a, b**).

Under W100, in both phenological stages, the mean RWC values of the plants subjected to chitosan concentrations did not show significant differences (*P* > 0.05) when compared to their respective controls in the absence of CHS (**Figures 2a, b**).

When analyzing electrolyte leakage (EL%) at the V5 phenological stage with water restriction and without chitosan, the plants did not show a significant difference (*P* > 0.05) compared to W100 without CHS (**Figure 2c**). Plants subjected to concentrations of 50 and 75 mg. L^-1^ of CHS, in W50, presented significant reductions of 32.3 and 28.8%, respectively, when compared in the absence of CHS. In contrast, in W100 and with the application of 50 and 75 mg L^-1^ of CHS, the plants did not present a significant difference (*P* > 0.05) in the EL (**Figure 2c**).

Under water restrictions and in the absence of CHS at the V7 phenological stage, the plants exhibited a significant increase of 33.5% compared to W100. Notably, at the same stage under the W50, plants treated with 50 and 75 mg L^-1^ of CHS showed reductions in this variable of 36.5% and 41.6%, respectively, compared to W50 without the attenuator. In W100, following the application of 50 and 75 mg L^-1^ of CHS, significant reductions in this variable were observed compared to the control without CHS (20.2% and 32.2%, respectively) (**Figure 2d**).

Reductions of 37.2% and 37.6% in WUE were observed at stages V5 and V7 under water restriction in the absence of CHS, respectively, compared to W100 without CHS (**Figures 2e, f**). In contrast, plants treated with 50 and 75 mg L^-1^ of CHS under water restriction at stage V5 showed increases of 109% and 59.4%, respectively, compared to untreated plants (**Figure 2e**). At stage V5 under W100, there were no significant differences (*P* > 0.05) between treatments. However, there were increases of 27.6% (50 mg ^-1^ of CHS) and 22.6% (75 mg ^-1^ of CHS) compared to the 100% water replacement depth (**Figure 2e**).

At the V7 stage, under 50% water replacement, applying 50 and 75 mg ^-1^ of CHS resulted in a 113% and 217.1% increase in WUE, respectively, compared to untreated plants. Under full water replacement (W100) at the V7 stage, plants treated with 75 mg ^-1^ of CHS exhibited a 65% increase in WUE compared to the control (without CHS) (**Figure 2f**).

In stage V5, under water restriction without CHS, lipid peroxidation (MDA) increased by 187.4% compared to W100 without CHS. However, plants showed lower MDA values at 50 mg L^−^¹ (73.5%) and 75 mg L^−^¹ (86.6%) compared to plants in W50 without CHS. In W100 at stage V5, there were no differences in MDA levels between different CHS concentrations compared to W100 (**Figure 2g**).

In the absence of CHS, cowpea plants exhibited higher MDA levels under water restriction (W50), showing a 204.9% increase compared to plants in W100 without CHS. Additionally, at stage V7 and under W50 conditions, a decrease of 17.3% in MDA levels (50 mg L^-1^ of CHS) and 49.2% (75 mg L^-1^ of CHS) was observed compared to plants without CHS (**Figure 2h**). When supplied with 100% water replacement and 75 mg L^-1^ of CHS, a reduction of 18.2% in MDA levels was noted at stage V7 compared to untreated plants (**Figure 2h**).

### 3.2 Leaf pigments

**Figure 3:**
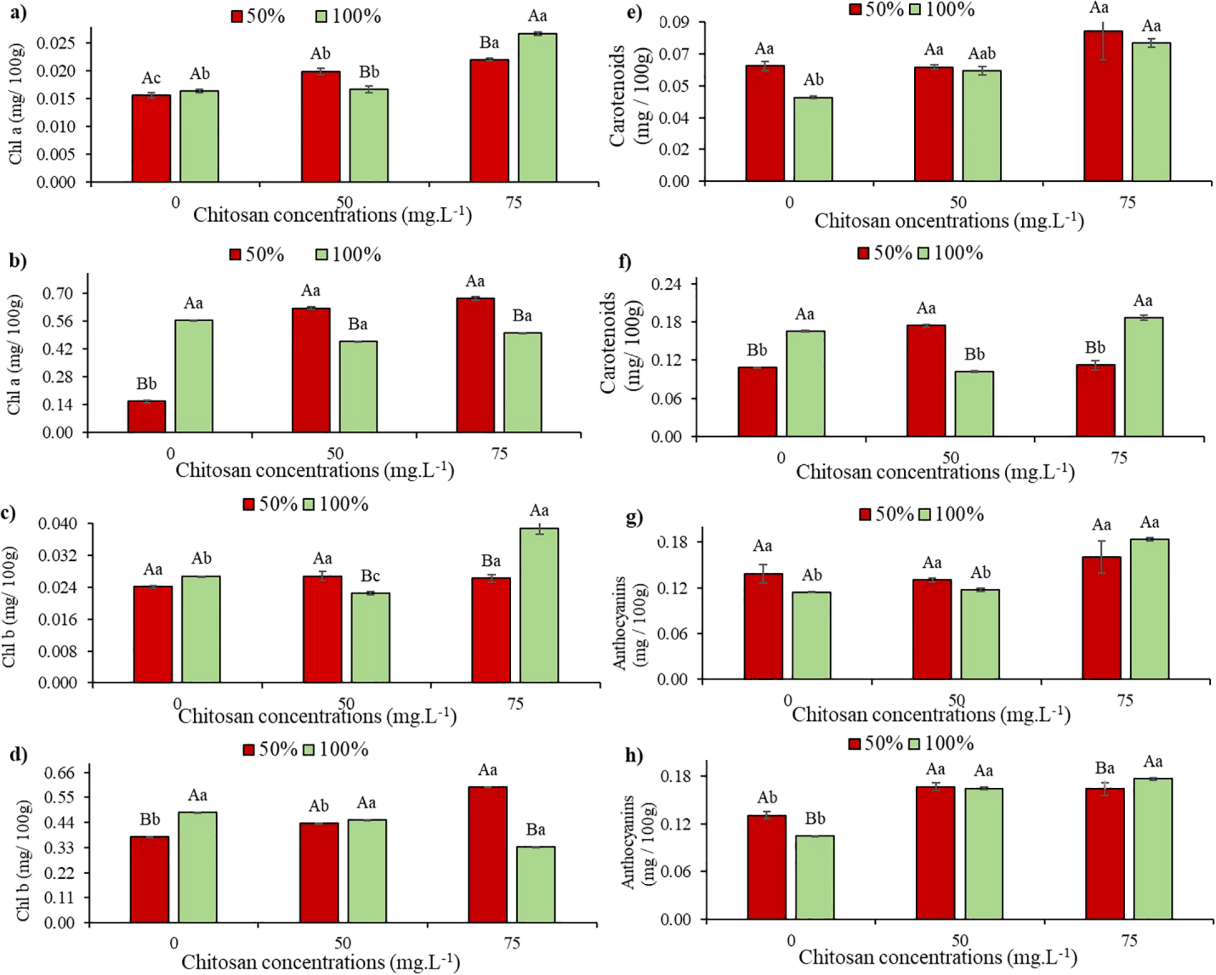
Chlorophyll a (Chl a) at phenological stages V5 **(a)** and V7 **(b)**; Chlorophyll b (Chl b) at phenological stages V5 **(c)** and V7 **(d)**; Carotenoids at phenological stages V5 **(e)** and V7 **(f)** and Anthocyanins at phenological stages V5 **(g)** and V7 **(h)** of cowpea cv. BRS Olhonegro, conditioned to two transparency slides (50% and 100% water configuration of crop evapotranspiration) and three chitosan concentrations (0, 50 and 75 mg. L^-1^). Secret letters differentiate the transparency slides within each chitosan concentration (t-Student *P* ≤ 0.05) and lowercase letters differentiate the chitosan concentrations within each irrigation slide (Tukey *P* ≤ 0.05).

At stage V5, chlorophyll a levels did not show a significant difference in plants under water deficit without CHS compared to those without CHS in W100 (**Figure 3a**). However, at V7 under the same condition, there was a 72.3% reduction in chlorophyll a levels in plants without CHS compared to W100 without CHS. In contrast, at V5 and W50, plants treated with 50 mg L^-1^ and 75 mg L^-1^ of CHS showed increases of 27.5% and 41%, respectively, compared to plants in W100 without CHS (**Figure 3a**). Additionally, at stage V5 under W100, there were increases of 1.84% (50 mg L^-1^ of CHS) and 63.8% (75 mg L^-1^ of CHS) in chlorophyll a levels compared to plants without the biopolymer (**Figure 3a**).

At the V7 stage and under W50, the plants showed a significant difference in Chl a (*P<* 0.05), with increases of 300.6% (50 mg L^-1^ of CHS) and 332% (75 mg L^-1^ of CHS) (**Figure 3b**). At W100, the plants had increases of 18% (50 mg L^-1^ of CHS) and 11.5% (75 mg L^-1^ of CHS) compared to plants without CHS (**Figure 3b**).

Under water restriction and in the absence of CHS (0 mg L^-1^), there was no significant difference (*P* > 0.05) in chlorophyll b contents in both stages V5 and V7 compared to W100 without CHS (**Figures 3c, d**). Similar results were observed in V5 under W50, where the application of 50 and 75 mg L^-1^ of CHS did not show significant differences (*P* > 0.05) compared to untreated plants (**Figure 3c**). However, under W100, chlorophyll b exhibited a 15.73% reduction (50 mg L^-1^ of CHS) and a 45.3% increase (75 mg L^-1^ of CHS) compared to plants without CHS treatment (**Figure 3c**).

At the V7 and W50 stages, following the application of 50 and 75 mg L^-1^ of CHS, there were increases of 16.2% and 58.2% in Chl b, respectively, compared to plants that did not receive CHS. At W100, Chl b values decreased by 6.81% (50 mg L^-1^ of CHS) and 30.9% (75 mg L^-1^ of CHS) compared to the control without CHS (**Figure 3d**).

At stage V5, under water restriction conditions and in the absence of CHS, there was no significant difference (*P* > 0.05) observed in carotenoid levels compared to W100 without CHS (**Figure 3e**). However, at stage V7 under the same experimental conditions, there was a 39.7% reduction in carotenoid levels compared to W100 (**Figures 3e, f**).

At stage V5 and under water restriction, plants treated with 75 mg L^-1^ of CHS showed a 29.9% increase in carotenoid content compared to untreated plants although the difference was not statistically significant (*P* > 0.05) (**Figure 3e**). At W100, plants treated with 75 mg L^-1^ of CHS exhibited a higher average carotenoid value of 0.0846 mg/100g compared to untreated plants (**Figure 3e**).

At stage V7 and under W50, plants treated with 50 mg L^-1^ of CHS showed a 60.5% increase in carotenoid content compared to untreated plants. At W100, plants sprayed with 75 mg L^-1^ of CHS exhibited a 12.6% increase in this pigment compared to untreated plants (**Figure 3f**).

At stage V5, without water restriction and CHS, there was no significant difference (*P* > 0.05) in the mean values of anthocyanin. However, an increase of 21% was observed compared to the same treatment in W100 (**Figure 3g**). Similar trends were observed at concentrations of 50 and 75 mg L^-1^ of CHS, with a 15.9% increase in this variable at the highest concentration (**Figure 3g**).

When anthocyanins were evaluated at stage V7 (**Figure 3h**) under water restriction without CHS, there was a 25.9% increase compared to W100 without CHS. However, when CHS was applied, increases of 27.4% and 25.2% (at 50 and 75 mg L^-1^, respectively) were observed compared to control irrigation without CHS. In W100, significant results were observed (*P* ≤ 0.05) with an increase of 57.7% (at 50 mg L^-1^ of CHS) and 69.2% (at 75 mg L^-1^ of CHS) compared to plants without CHS (**Figure 3h**).

### 3.3 Antioxidant mechanism activity

**Figure 4:**
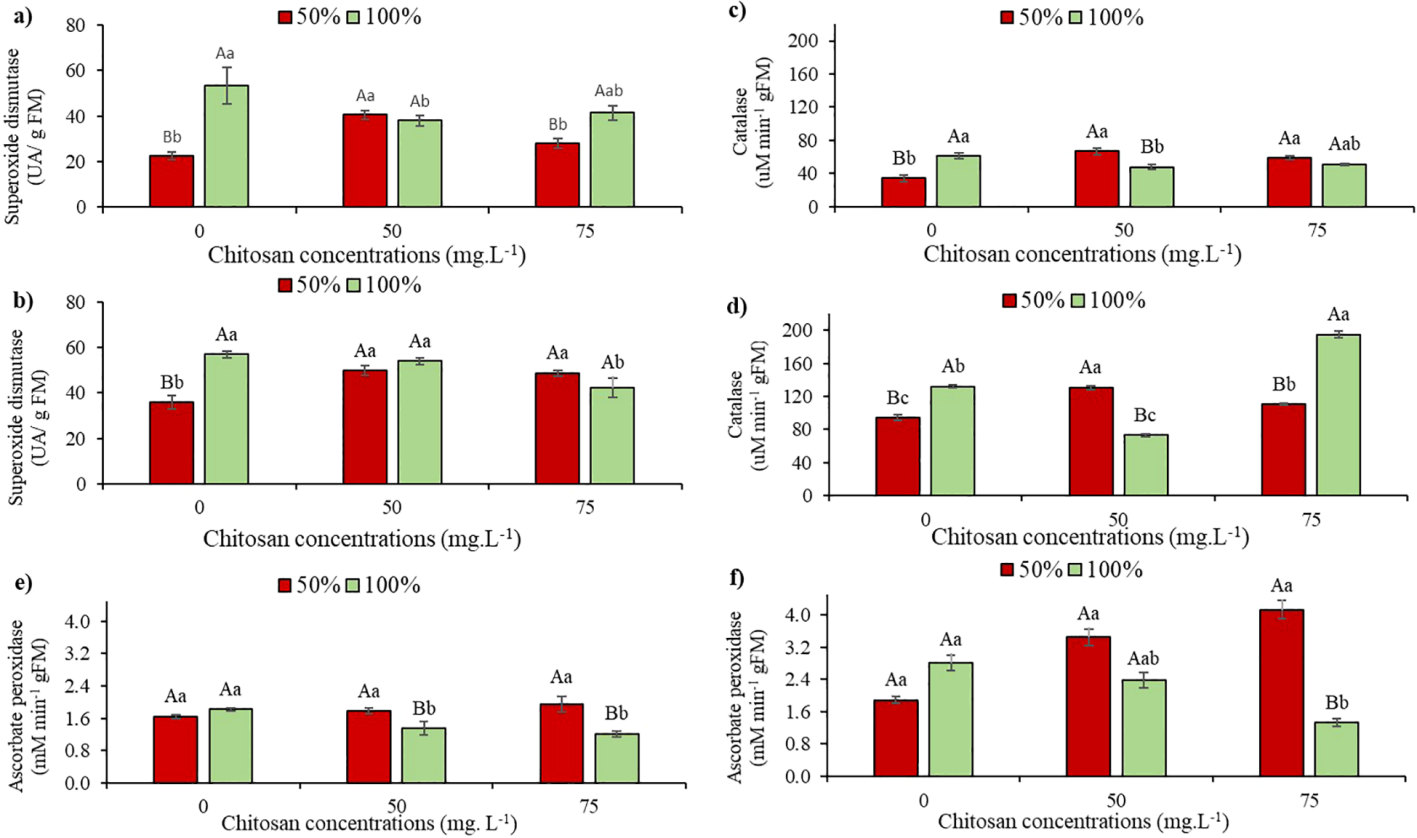
Superoxide Dismutase activity at phenological stages V5 **(a)** and V7 **(b)**; catalase activity at phenological stages V5 **(c)** and V7 **(d)**; and ascorbate peroxidase activity at phenological stages V5 **(e)** and V7 **(f)** of cowpea cv. BRS Olhonegro, conditioned to two irrigation depths (50% and 100% water replacement from crop evapotranspiration) and three chitosan concentrations (0, 50, and 75 mg. L^-1^). Capital letters differentiate the irrigation depths within each chitosan concentration (t-Student *P* ≤ 0.05) and lowercase letters differentiate the chitosan concentrations within each irrigation depth (Tukey *P* ≤ 0.05).

When analyzing the activity of the superoxide dismutase (SOD) enzyme at the V5 stage, under water restriction and in the absence of CHS, a 58% reduction was observed compared to W100 (0 mg. L^-1^). In this same water condition, an 80.4% increase in SOD activity was observed when 50 mg. L^-1^ of CHS was applied, compared to the control treatment (**Figure 4a**). With 100% water replacement, the plants showed a 28.7% decrease in SOD activity after the application of 50 mg L^-1^ of CHS. However, with 75 mg L^-1^ of CHS, the reduction was 21.8% compared to the control under the same irrigation condition (**Figure 4a**).

At stage V7, under water restriction without CHS, a significant 37% reduction in SOD activity was observed compared to W100. Following CHS application, there were increases of 38.6% (50 mg L^-1^ of CHS) and 35.5% (75 mg L^-1^ of CHS) in W50 compared to W100 and in the absence of CHS (**Figure 4b**). In W100 with the application of 75 mg L^-1^ of CHS, there was a 26.3% increase in SOD activity compared to plants without CHS application (**Figure 4b**).

Regarding the activity of the catalase enzyme (CAT), at the V5 stage, a reduction of 44% was observed due to the suppression of water availability and in the absence of CHS. For plants that received CHS (50 and 75 mg. L^-1^), CAT activity increased by 95.3% and 72.1%, respectively, compared to plants that did not receive CHS (**Figure 4c**). At W100, plants sprayed with CHS did not differ (**Figure 4c**).

Under water restriction and without CHS application, a 28.6% reduction in CAT activity was observed at stage V7 compared to the control treatment (**Figure 4d**). Under water restriction and without CHS application, a 28.6% reduction in CAT activity was observed at stage V7 compared to its control treatment (**Figure 4d**). Additionally, plants subjected to water restriction showed a 39% increase in CAT activity with 50 mg L^-1^ of CHS and a 16.8% increase with 75 mg L^-1^ of CHS compared to plants without CHS application (**Figure 4d**). Consequently, at W100, CAT activity was enhanced in plants treated with 75 mg L^-1^ of CHS (**Figure 4d**).

At the V5 phenological stage, in the absence of CHS, the mean values showed no significant difference (*P* > 0.05) among the irrigation depths in ascorbate peroxidase (APX) activity. Even after applying 75 mg L^-1^ concentration of CHS to plants under water restriction, there was no significant difference (*P* > 0.05) compared to plants without CHS under the same water conditions. In the W100 treatment, there was a significant increase (*P* ≤ 0.01) of 16.5% (50 mg L^-1^ of CHS) compared to plants not treated with CHS (**Figure 4e**).

When APX activity was evaluated at the V7 stage under water restriction in the absence of QS, the plants exhibited a 33% reduction compared to their respective control blade. However, when QS was applied at concentrations of 50 and 75 mg L^−^¹, increases of 82% and 118.5% were observed, respectively, compared to the treatment without chitosan (**Figure 4f**). Furthermore, at the V7 stage under W100, it was observed that after the plants were conditioned to a concentration of 75 mg L^−^¹ QS, APX activity decreased by 52.6% (*P* ≤ 0.01) (**Figure 4f**).

### 3.4 Osmotic adjustment mechanism

**Figure 5:**
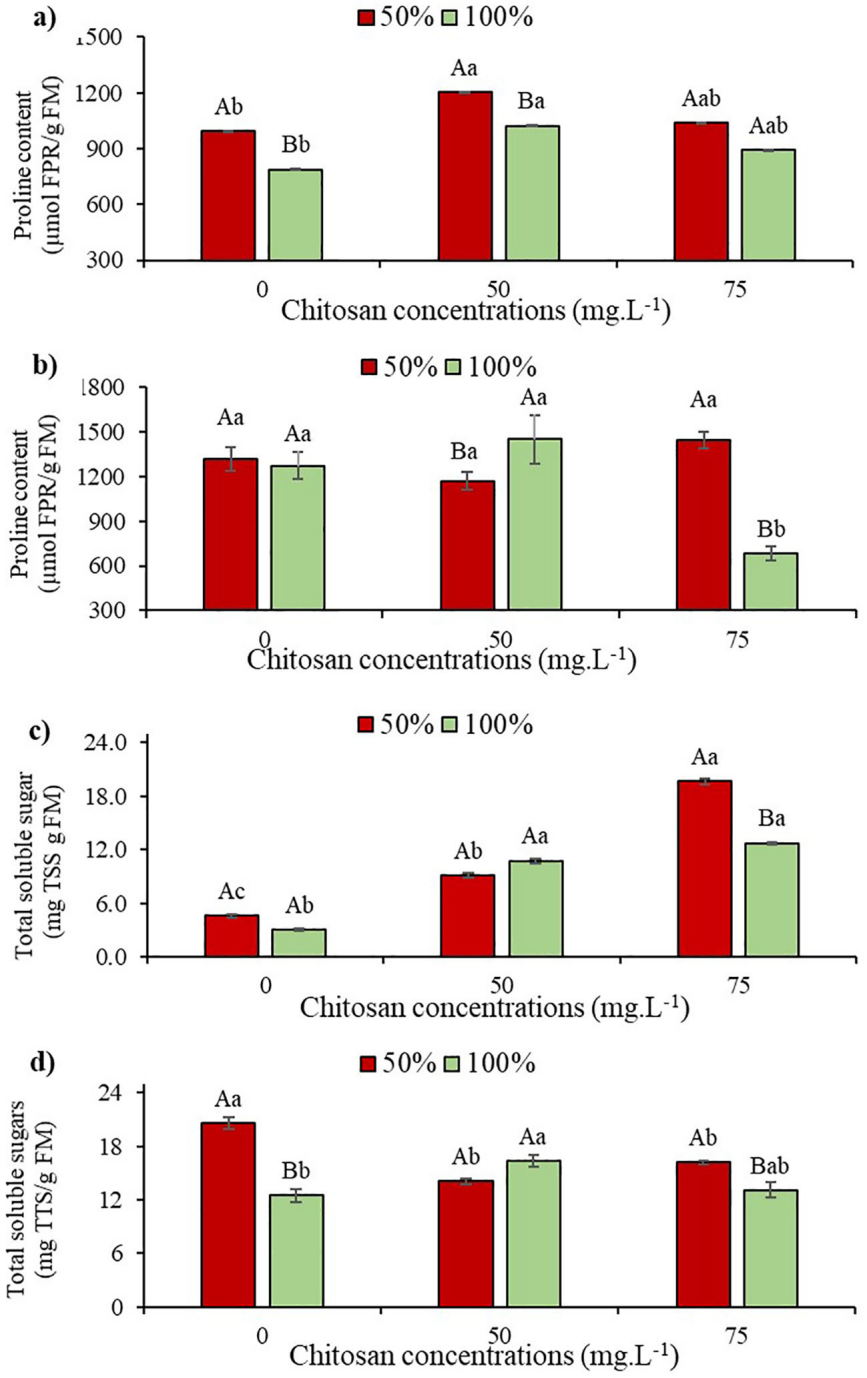
Proline content at phenological stages V5 **(a)** and V7 **(b)** and total soluble sugars at phenological stages V5 **(c)** and V7 **(d)** of cowpea cv. BRS Olhonegro, conditioned to two irrigation depths (50% and 100% water replacement from crop evapotranspiration) and three chitosan concentrations (0, 50, and 75 mg. L^-1^). Capital letters differentiate the irrigation depths within each chitosan concentration (t-Student *P* ≤ 0.05) and lowercase letters differentiate the chitosan concentrations within each irrigation depth (Tukey *P* ≤ 0.05).

It was observed that in the absence of CHS, plants subjected to water restriction showed a 26% increase in free proline (FPR) content compared to the control treatment at stage V5 (**Figure 5a**). However, after applying 50 mg L^-1^ to the plants under water stress, there was a 21.3% increase in proline content in W100 at stage V5 (**Figure 5a**).

No significant difference (*P* > 0.05) was observed in irrigation depths at phenological stage V7. However, in the absence of CHS, there was a 3.69% increase in FPR content (**Figure 5b**). At stage V7 in W50, there was no significant difference (*P* > 0.05) in CHS concentrations, but with 75 mg L^-1^ of CHS, there was a 9.57% gain in FPR compared to W100 without CHS (**Figure 5b**). When plants received 100% water replacement at stage V5, a 29.9% increase in proline content was observed with the application of 50 mg L^-1^ of CHS compared to plants without this biopolymer (**Figure 5b**).

In plants at stage V5, there were no significant differences (*P* > 0.05) in total soluble sugar (TSS) content among different irrigation depths when CHS was absent. However, under water restriction, there was a 52% increase in TSS contente compared to W100 without CHS (**Figure 5c**). At this stage and with W50 irrigation, plants treated with CHS showed a significant increase of 99.5% (50 mg L^-1^ of CHS) and 332% (75 mg L^-1^ of CHS) in TSS content compared to plants without CHS at the same irrigation depth (**Figure 5c**). For W100, there was a variation in TSS content with higher values observed in plants treated with CHS, indicating increases of 256.6% (50 mg L^-1^ of CHS) and 323.3% compared to those without CHS at the same W100 irrigation depth (**Figure 5c**).

At stage V7 with 50% water replacement, there was a 64.8% increase in TSS content compared to plants without CHS in W100 (**Figure 5d**). At this stage with W50, following treatment with CHS (50 and 75 mg L^-1^), reductions of 32% and 17.9% were observed, respectively, compared to W100 without CHS (**Figure 5d**). Under 100% water replacement at stage V7, there was a 30.4% increase in TSS content after applying 50 mg L^-1^ of CHS, compared to the control (without CHS) (**Figure 5d**).

### 3.5 Growth and dry mass gain indicators

**Figure 6:**
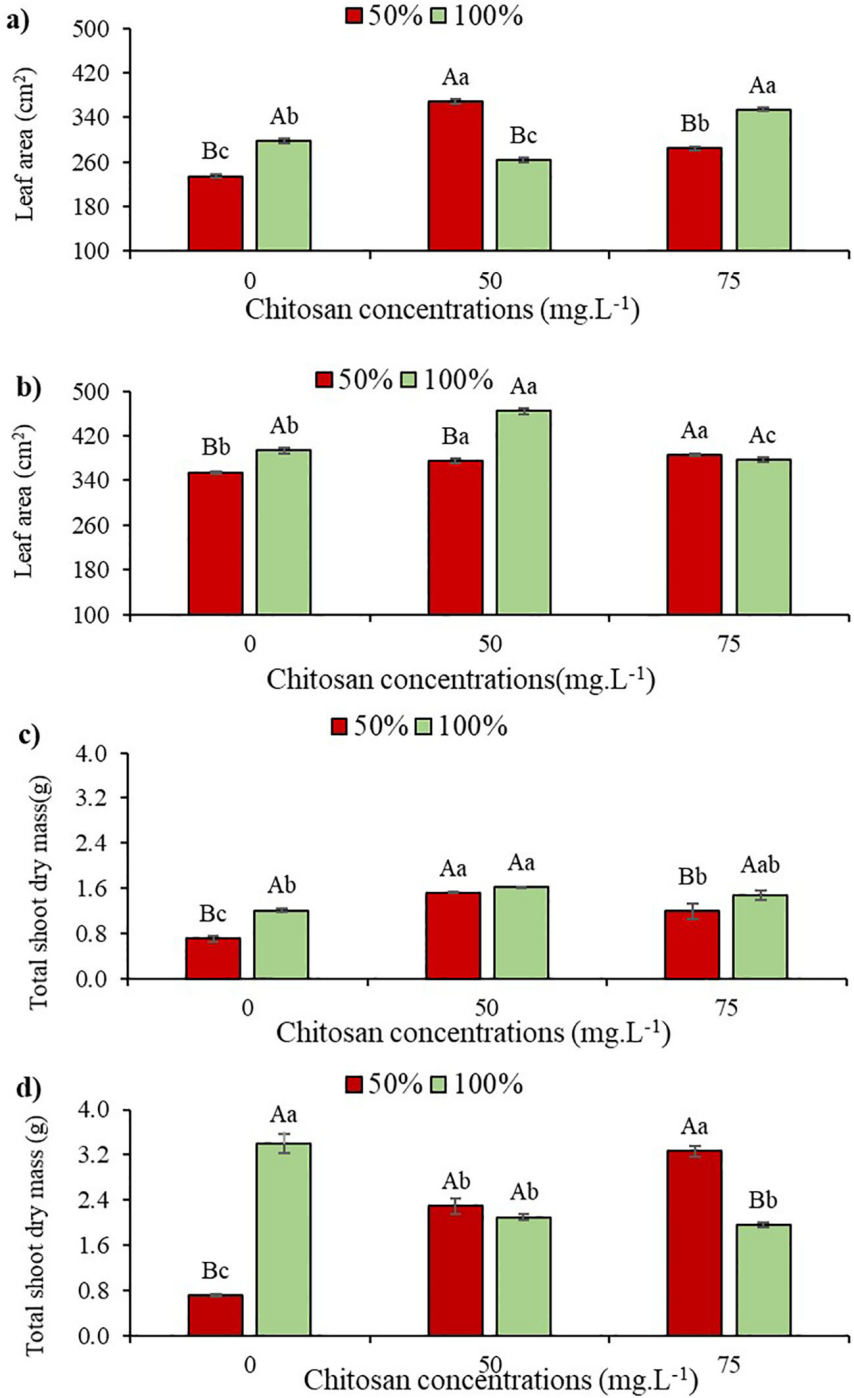
Leaf Area at phenological stages V5 **(a)** and V7 **(b)** and Total Dry Mass of the Aerial Part at phenological stages V5 **(c)** and V7 **(d)** of cowpea cv. BRS Olhonegro, conditioned to two irrigation depths (50% and 100% water replacement from crop evapotranspiration) and three chitosan concentrations (0, 50 and 75 mg. L^-1^). Capital letters differentiate the irrigation depths within each chitosan concentration (t-Student *P* ≤ 0.05) and lowercase letters differentiate the chitosan concentrations within each irrigation depth (Tukey *P* ≤ 0.05).

In the absence of CHS and under water deficit conditions (W50), total leaf area (TLA) reduced by 21.4% and 9.9% at phenological stages V5 and V7, respectively, when compared to the control treatment (W100) (**Figures 6a, b**).

At stage V5 and under water restriction, TLA increased by 57.16% and 21.54% with 50 and 75 mg. L^-1^ of CHS, respectively, compared to W50 without CHS. At this same stage and under W100, there were gains of 18% and 18.86% in TLA at concentrations of 50 and 75 mg. L^-1^ of CHS, respectively, compared to the control treatment (**Figure 6a**).

In V7 and under W50, applying 50 and 75 mg L^-1^ of CHS to plants induced increases of 5.9% and 8.8% in LA compared to the control treatment (0 mg L^-1^). In W100, foliar spraying with 50 mg L^-1^ of CHS led to an 18% increase in TLA compared to plants without CHS application (**Figure 6b**).

At the V5 stage, cowpea plants exhibited a 9.5% reduction in total shoot dry mass under water restriction without CHS application. Conversely, when subjected to water restriction and treated with 50 and 75 mg L^-1^ of CHS, TSDM increased by 42.8% and 14.3%, respectively, compared to plants without CHS. This tendency was consistent even under 100% water replacement, where the application of 50 mg L^-1^ of CHS resulted in a 37.9% increase in TSDM compared to the control treatment without CHS (**Figure 6c**).

At the V7 phenological stage, a 79% reduction in TSDM was observed without CHS application under W50 compared to 100% water replacement depth without CHS. However, in V7 under W100, with 50 and 75 mg L^-1^ of CHS, there was a reduction in TSDM of 222.3% and 358.56%, respectively, compared to plants not treated with this biopolymer (**Figure 6d**).

### 3.6 Pearson’s correlation matrix for the treatments under water restriction and chitosan

**Figure 7:**
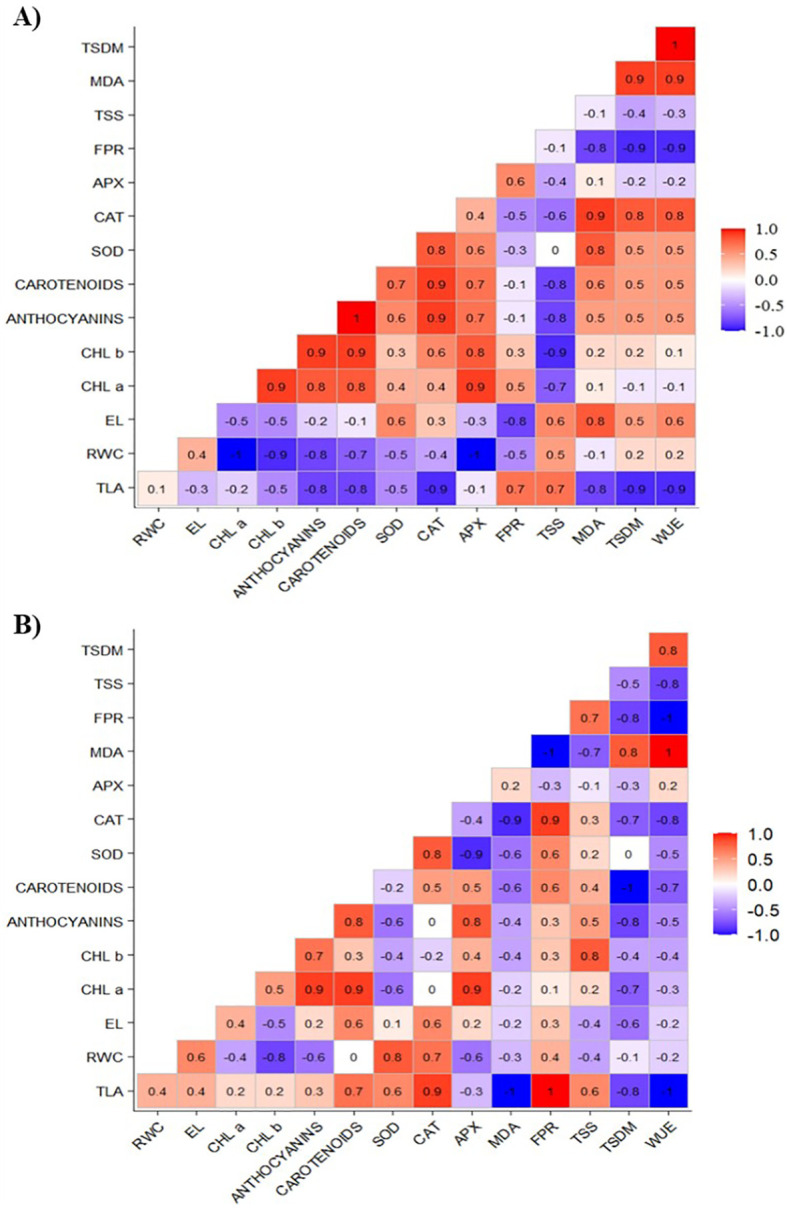
Correlation matrix of total leaf area (TLA), relative water content (RWC), electrolyte leakage (EL), chlorophyll a (Chl a), chlorophyll b (Chl b), carotenoids, anthocyanins, superoxide dismutase (SOD), catalase (CAT), ascorbate peroxidase (APX), free proline content (FPR), total soluble sugars (TSS), lipid peroxidation (MDA), total shoot dry mass (TSDM), and water use efficiency (WUE) of cowpea cv. BRS Olhonegro under water restriction (50% of ETc) at a chitosan concentration of 50 mg L^-1^, and phenological stages V5 **(A)** and V7 **(B)**.

**Figure 7A** illustrates a positive correlation between the increase in growth and total shoot dry mass gain (TLA and TSDM) with osmotic adjustment mechanisms (FPR and TSS) and antioxidant mechanisms (CAT). Additionally, CAT exhibited a positive correlation with the SOD enzyme and chloroplast pigments (carotenoids and anthocyanins).

At the V7 stage (**Figure 7B**), TLA exhibited a positive correlation with carotenoids, CAT, and FPR. The enzyme CAT also showed positive correlation with RWC, SOD, and FPR. In contrast, the variables FPR and CAT were negatively correlated with MDA.

**Figure 8:**
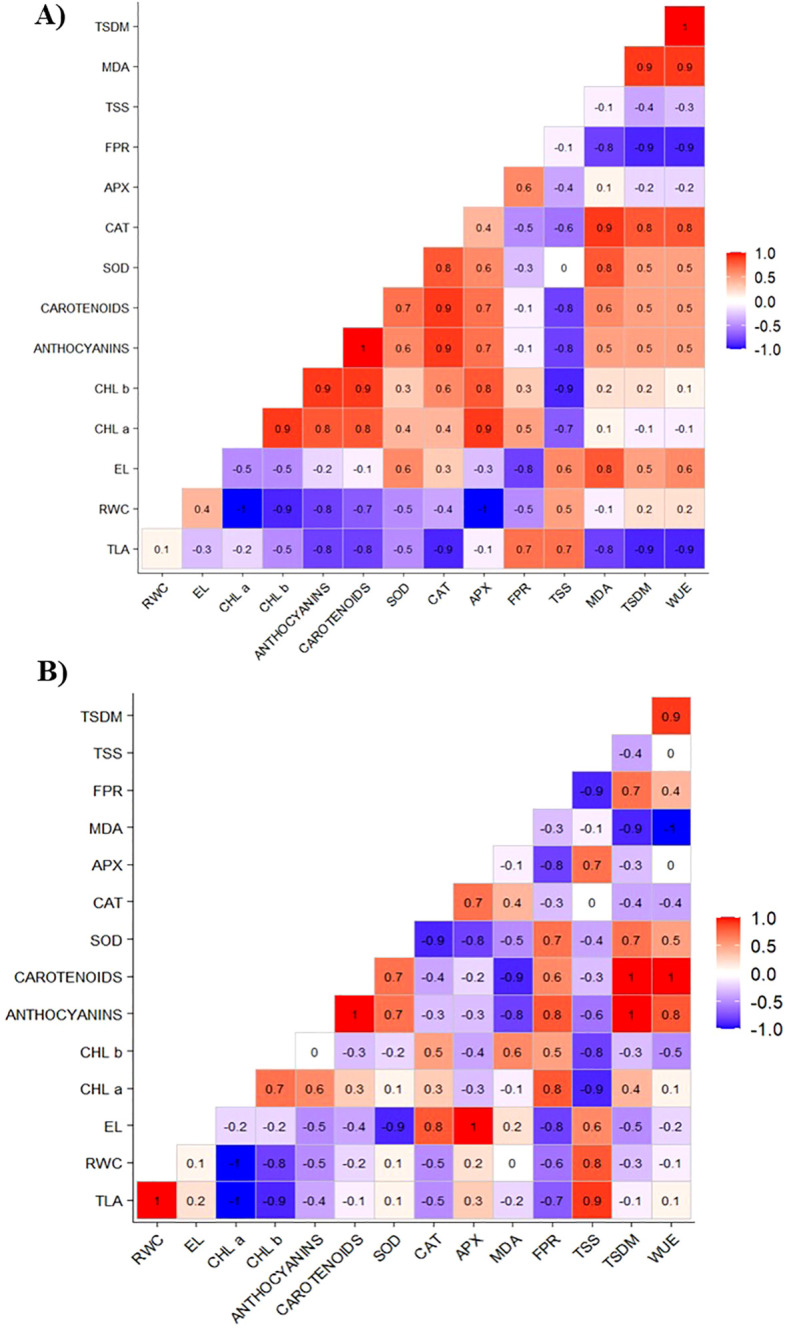
Correlation matrix of total leaf area (TLA), relative water content (RWC), electrolyte leakage (EL), chlorophyll a (Chl a), chlorophyll b (Chl b), carotenoids, anthocyanins, superoxide dismutase (SOD), catalase (CAT), ascorbate peroxidase (APX), free proline content (FPR), total soluble sugars (TSS), lipid peroxidation (MDA), total dry mass (TSDM), and water use efficiency (WUE) of cowpea cv. BRS Olhonegro under water restriction (50% of ETc) at a chitosan concentration of 75 mg L-1 and phenological stages V5 **(A)** and V7 **(B)**.

Applying 75 mg L^−^¹ of CHS under water restriction conditions, as illustrated in **Figure 8A**, demonstrates that the increase in the TLA and TSDM variables is positively correlated with the osmotic adjusters (FPR and TSS) and with CAT. In addition, CAT is correlated with the chloroplast pigments (carotenoids and anthocyanins), SOD, and MDA.

The increase in TLA showed a positive correlation with RWC and TSS (**Figure 8B**). Similarly, TSDM showed a strong positive correlation with the variables anthocyanins, carotenoids, SOD, and FPR. In addition, the variable WUE correlated with anthocyanins and carotenoids. Similarly, RWC, CAT, and FPR positively correlated with TSS, APX, and EL, respectively.

## 4 Discussion

### 4.1 Indicators of water *status* and membrane damage

In the present study, water restriction significantly impacted the water status of plants, leading to a decrease in relative water content (**Figures 2a, b**) and an increase in electrolyte leakage (**Figures 2c, d**), which inhibited plant growth. Cellular Relative Water Content serves as a reliable indicator of cellular and tissue hydration levels, crucial for optimal growth and physiological function (Silva et al., 2024). Increased electrolyte leakage indicates membrane damage due to water restriction (Silva et al., 2024).

At both CHS concentrations and under drought, plants showed an increase in RWC (**Figures 2a, b**) as well as reductions in EL (**Figures 2c, d**). Therefore, chitosan has a significant role in regulating osmosis during water restriction, and its application may be able to expand the cell layer. It improves the increase in membrane stability, which preserves the relative water content, as well as reducing membrane damage, as observed by Hassnain et al. (2020) and Abdelaal et al. (2021), when studying the efficacy of chitosan in tomato (*Lycopersicon esculentum* L.) and garlic (*Allium sativum* L.) under water restriction, respectively.

Water use efficiency can be defined as the amount of carbon assimilated in biomass or grains produced per unit of water consumed by the crop (Kocięcka and Liberacki, 2021). In this study, the decrease in WUE (**Figures 2e, f**) observed under water restriction in both stages of plant growth may be attributed to the impact of reduced TSDM in these water conditions. These findings are consistent with previous studies Zhao et al. (2020) that reported significant decreases in WUE in wheat plants (*Triticum aestivum* L.) grown under moderate and severe water stress.

In this study, an increase in WUE was observed with both chitosan concentrations and water conditions. This finding supports previous research that showed enhanced WUE in plants treated with CHS under well-irrigated and drought conditions (Farouk and El-Metwally, 2019). The improved WUE may be due to the effects of CHS on leaf pigment biosynthesis, osmotic adjustment, and antioxidant capacity.

It is important to emphasize that cowpea plants show increased lipid peroxidation (MDA) under water restriction, indicating oxidative stress (**Figures 2g, h**), primarily caused by hydrogen peroxide production. Cowpea genotypes that are more susceptible to stress exhibit higher levels of MDA compared to the more tolerant ones (Carvalho et al., 2019; Melo et al., 2022).

The concentrations of CHS reduced MDA levels in cowpea plants under water restriction (**Figures 2g, h**). This is attributed to the enhanced antioxidant activity that eliminates ROS and shields cell membranes from oxidative stress (Hawrylak-Nowak et al., 2021). Chitosan triggers the activation of SOD and CAT, which play a crucial role in eliminating H_2_O_2_ in plants. Additionally, antioxidant production reduced free radical formation and peroxidation during stress (Dawood et al., 2024). Furthermore, charge-charge interactions between positively charged chitosan amine groups and negatively charged membrane phospholipids promote a signal leading to the octadecanoid pathway (Almeida et al., 2020).

### 4.2 Leaf pigments

Although there was no significant difference in chlorophyll content (**Figures 3a–d**), it is important to mention that water restriction can lead to its decline, which results in a decrease in the opening of stomata to prevent water loss through transpiration, which causes a reduction in the absorption of atmospheric CO_2_ essential for photosynthesis (Ezin et al., 2021). Similar to the current study, Amir et al. (2021) observed that water restriction significantly increased the accumulation of anthocyanins. These pigments serve as powerful antioxidants and are essential for detoxifying harmful oxidants generated during stressful conditions (Manoj et al., 2022).

Under water restriction, plants treated with CHS showed an increase in leaf pigments such as carotenoids (**Figures 3e, f**) and anthocyanins (**Figures 3g, h**). This response may be associated with the role of CHS in improving cytokinin contents that stimulate chlorophyll synthesis and/or in increasing the availability of amino compounds released from CHS (Dawood et al., 2024).

The increase in anthocyanins in this study confirms previous findings by Manoj et al. (2022), who reported a rise in this compound in red beans (*Phaseolus vulgaris* L.) treated with 0.25% chitosan and exposed to water restriction. Anthocyanins are crucial in plant responses, particularly under stress conditions. They serve as eliminators of reactive oxygen species (ROS), offering photoprotection and acting as stress indicators. This versatility may explain the strong antioxidant capacity observed in plants with elevated anthocyanin levels under water stress conditions reported by Manoj et al. (2022).

### 4.3 Antioxidant mechanism activity

In response to water deficit that causes oxidative stress, plants typically increase the activity of the enzymes SOD (**Figures 4a, b**), CAT (**Figures 4c, d**), and APX (**Figures 4e, f**) in an attempt to neutralize and eliminate ROS, thus coordinating a defensive repertoire against oxidative damage (Xiong et al., 2018). Illustratively, SOD plays an essential role as an enzyme, promoting the conversion of superoxide anions (O_2_^-^) into molecular hydrogen peroxide (H_2_O_2_) through redox reactions. In contrast, APX acts as a fundamental component by facilitating the transformation of H_2_O_2_ into water, using ascorbic acid as a reducing agent. Simultaneously, CAT catalyzes the decomposition of H_2_O_2_ into water and oxygen, thereby neutralizing its potential harmful effects (Melo et al., 2022; Alencar et al., 2024; Silva et al., 2024).

According to the current investigation, cowpea plants, under the blade with 50% water replenishment, expressed reductions in the antioxidant activity of all tested enzymes. This change implies that the studied cultivar may involve alternative tolerance mechanisms, in addition to the immediate and positive regulation of antioxidant enzymes, thus highlighting the multifaceted adaptive strategies used in mitigating the effects of oxidative stress caused by water deficit (Cavalcante et al., 2024; Silva et al., 2024). The increases in antioxidant enzymes observed in the present study after the use of chitosan in plants subjected to water stress may be due to the crucial role of chitosan in this stress defense mechanism, acting as an antioxidant so that ROS can react with the OH group and the amino group at positions C-2, C-3, and C-6 of the pyranose ring to produce a stable macromolecule (Muthu et al., 2021). Additionally, chitosan induces increases in antioxidant enzymes, such as SOD, CAT, and APX, which mitigate the adverse effects of water stress by enhancing water absorption through root growth, reducing the accumulation of free radicals, and improving the photosynthetic apparatus (Hidangmayum and Dwivedi, 2023).

### 4.4 Osmotic adjustment mechanism

It is important to observe that cowpea plants have the ability to accumulate osmoprotective molecules such as FPR and TSS (Silva et al., 2024). These molecules play a crucial role in enhancing water absorption, particularly in environments with limited water availability, and are vital for plants to withstand water stress (Santos et al., 2022; Jales-Filho et al., 2022; Silva et al., 2024). The study revealed significant increases in FPR (**Figures 5a, b**) and TSS (**Figures 5c, d**) contents.

Under water-deficient conditions, the concentration of TSS is altered in plant tissues, affecting the translocation efficiency of photoassimilates and influencing plant development and respiration (Moura et al., 2016). Previous studies have highlighted the importance of these organic solutes in maintaining plant survival and contributing to osmotic adjustment (Martins et al., 2024).

Plants under CHS application and water restriction showed a significant increase in proline concentration when compared to the control (**Figures 5a, b**). This increase could be attributed to the synthesis of covalent bonds between proline and the amino group released by CHS, and may also have been the result of the bonds between the CHS hydroxyl group and the proline carboxyl group, providing stability between the molecules (Thambiliyagodage et al., 2023). Thus, the function of proline as an osmoprotector becomes evident, given that it helps to balance the osmotic potential between cells and their surroundings (Ibrahim et al., 2023). Previous studies have also demonstrated the importance of proline in safflower plants (*Carthamus tinctorius* L.) coping with water scarcity by enhancing osmotic adjustment, thereby supporting turgor pressure and stomatal conductance under water-deficient conditions (Abdiazar et al., 2024).

For TSS (**Figures 5c, d**), plants treated with CHS showed substantial increases in sugars, emphasizing the importance of these compounds in mitigating the negative effects of water restriction. This report corroborates the data from Bakhoum et al. (2022) and Bakry et al. (2024), who observed increases in the content of total soluble sugars in *Lupinus termis* L. and *Arachis hypogea* L. plants under drought conditions, where using chitosan proved to be extremely important. This effect can be attributed to the impact of this biopolymer on the activity of the photosynthetic pigments of these species, which results in a gain in the transport of sugars through the leaves.

### 4.5 Growth and dry mass gain indicators

Water restriction is a significant abiotic stress factor that inhibits plant growth and results in various physiological and biochemical changes (Bakhoum et al., 2022). In this study, water restriction negatively impacted the total leaf area (TLA) (**Figures 6a, b**) and total shoot dry mass (TSDM) (**Figures 6c, d**) of cowpea plants. The decrease in TLA and TSDM due to water stress may be linked to disruptions in physiological and biochemical processes, such as the regulation of phytohormones, photosynthetic assimilation, and the activity of key enzymes involved in metabolic pathways (Dawood et al., 2024). On the other hand, the application of CHS alleviated the effects of water restriction on the growth attributes of cowpea (**Figures 6a–d**). These effects can be attributed to the improvement in the absorption of water and essential nutrients by plants, which may be associated with chitosan’s ability to stimulate the development and growth of the root system (Farouk and El-Metwally, 2019), although this mechanism is not yet fully understood.

Furthermore, it is important to highlight that chitosan can stimulate the synthesis of hormones responsible for plant development and growth, such as salicylic acid (SA) and jasmonic acid (JA) (Suarez-Fernandez et al., 2020), and thus contribute to the maintenance of growth under stress conditions. However, under ideal water conditions, stimulating the production of these hormones can lead to a false alert and divert resources that would be used in growth to activate these responses (Borborema et al., 2025), which may explain the reductions in dry mass observed in the present study under the condition of 100% water replacement.

### 4.6 Chitosan enhances cowpea growth through osmotic and antioxidant adjustment

In **Figure 7A**, the application of 50 mg L^-1^ of chitosan resulted in increased growth, as measured by TLA and TSDM. This growth was positively correlated with indicators of osmotic adjustment, specifically proline (FPR) and total soluble sugars (TSS). These findings can be attributed to the fact that higher levels of FPR and TSS improve cell turgor and enhance the plant’s defense mechanisms against reactive oxygen species (ROS) (Dawood et al., 2024). Consequently, this leads to overall improved plant growth, evidenced by the increases in TLA and TSDM.

Furthermore, the increases in antioxidant mechanisms are positively correlated with TLA and TSDM, highlighting the importance of reactive oxygen species (ROS) elimination for growth maintenance. Notably, catalase (CAT) and superoxide dismutase (SOD) showed a positive association with anthocyanins and carotenoids, emphasizing the integrated role of these antioxidants in protecting against oxidative stress (Manoj et al., 2022; Hidangmayum and Dwivedi, 2023).

At stage V7, there is a positive correlation among TLA, carotenoids, CAT, and FPR, which highlights the significance of chitosan application for photoprotection and osmoregulation in cowpea, ultimately benefiting the growth of this crop (**Figure 7B**). Additionally, CAT is positively correlated with RWC, SOD, and FPR, indicating that these components work together to alleviate oxidative and osmotic stress (Hidangmayum and Dwivedi, 2023). Additionally, the negative correlations observed between FPR and CAT with MDA suggest that higher levels of FPR and CAT may be linked to decreased lipid peroxidation, as elevated MDA levels indicate damage to cell membranes (Dawood et al., 2024). Similar findings were reported by other researchers (Ibrahim et al., 2023; Dawood et al., 2024), who noted increases in osmotic adjustment and antioxidant levels, as well as a reduction in MDA when cultivating *Vicia faba* and *Lactuca sativa* L. under treatments with chitosan and water restriction.

The application of 75 mg L^−^¹ of CHS under water restriction, as shown in **Figure 8A**, leads to enhanced positive effects on growth and defense mechanisms. The positive correlation observed among TLA, TSDM, osmotic adjusters, and CAT suggests that CHS promotes a more effective adaptive response, thereby strengthening both antioxidant and osmotic defense mechanisms (Dawood et al., 2024). CAT plays a crucial role in the antioxidant system of plants and shows a positive correlation with carotenoids, anthocyanins, and SOD. Together, they form a defense network against ROS. The interaction between CAT and SOD, an essential enzyme in the initial stage of antioxidant defense, indicates a cooperative effort in eliminating ROS. SOD converts superoxide into hydrogen peroxide, which is then broken down by CAT. This process helps prevent cellular damage, also caused by MDA (Hawrylak-Nowak et al., 2021; Dawood et al., 2024).

CHS plays a vital role in enhancing water retention and promoting the synthesis of soluble sugars (**Figure 8B**), which are essential for plant survival during stressful conditions. The Strong correlation between TDSM and the enzymes SOD and FPR highlights the importance of CHS in this mechanism (Ibrahim et al., 2023). By modulating the levels of FPR and SOD, CHS helps improve protection against oxidative stress by stabilizing the plasma membrane and eliminating reactive oxygen species (ROS) (Abdelaal et al., 2021).

These results highlight the substantial benefits of using chitosan in cowpea cultivation, which may encourage the use of this product in similar applications. These facts, associated with the non-toxic, biocompatible and biodegradable nature of chitosan (Alenazi et al., 2024), make this product an interesting alternative for sustainable agriculture when used alone. Since, according to Bandara et al. (2020), depending on the formulation, compounds added to chitosan, such as metals, for example, can accumulate in soil, water and biological systems, causing damage to the environment.

## 5 Conclusions

Chitosan demonstrated effectiveness at both concentrations tested. At the V5 development stage, the 50 mg L^-1^ concentration positively influenced relative water content, superoxide dismutase enzyme activity, proline content, and total shoot dry mass. Additionally, it reduced the leakage of intracellular electrolytes. At the V7 stage, a concentration of 75 mg L^-1^ was particularly effective in lowering the impact of water restriction, primarily by enhancing the activity of ascorbate peroxidase, proline, and chlorophyll b in the BRS Olhonegro cultivar. This cultivar showed a high response to foliar application of the biopolymer. We can conclude that the foliar application of chitosan mitigated the harmful effects of water stress in cowpea plants by maintaining water homeostasis, improving photosynthetic pigments, activating antioxidant mechanisms, and providing osmoprotection for the crop.

## Data Availability

The original contributions presented in the study are included in the article/supplementary material. Further inquiries can be directed to the corresponding author.
